# Modified Laparoscopic Transabdominal Cervicoisthmic Cerclage for the Surgical Management of Recurrent Pregnancy Loss due to Cervical Factors

**DOI:** 10.3390/jcm10040693

**Published:** 2021-02-10

**Authors:** Hyewon Chung, Seungmee Lee, Changho Song, Tae-Kyu Jang, Jin-Gon Bae, Sang-Hoon Kwon, So-Jin Shin, Chi-Heum Cho

**Affiliations:** 1 Department of Obstetrics and Gynecology, Keimyung University School of Medicine, Daegu 42601, Korea; hyewonny81@naver.com (H.C.); seungmeemd@gmail.com (S.L.); songchanghomd@gmail.com (C.S.); tiber0103@naver.com (T.-K.J.); jgonmd@gmail.com (J.-G.B.); ksh1999@dsmc.or.kr (S.-H.K.); 2 Comprehensive Care Center for High Risk Pregnancy and Newborn, Keimyung University Dongsan Hospital, Daegu 42601, Korea

**Keywords:** recurrent pregnancy loss, uterine cervical incompetence, premature birth, cervical cerclage, laparoscopy

## Abstract

This study aimed evaluate the feasibility of modified laparoscopic transabdominal cervicoisthmic cerclage (LTCC) and its impact on recurrent pregnancy loss (RPL) and is a retrospective observational cohort study of patients who underwent modified LTCC from 2003 to 2018 (n = 299). The surgery was performed at a mean gestational age of 12.5 weeks (range 10.5–17.5 weeks). Of the 299 patients, 190 were reported as having undergone abortion (one abortion: 91 (47.9%), two: 59 (31.1%), three or more: 40 (21.1%)) before the present pregnancy and prior to the surgery. The mean operation time was 47.4 min (range 15–100 min). We followed up with 205 of 299 patients and recorded their obstetric outcomes. There were 176 successful deliveries via cesarean section, and the fetal survival rate was 85.9% (176/205). The results of this study suggest that modified LTCC is a safe and feasible surgical option during pregnancy for patients with a history of RPL due to cervical factors.

## 1. Introduction

The psychological impact of pregnancy loss is well known [1], and the loss of a wanted pregnancy at any stage is distressing, especially if it is a part of a pattern of recurrent pregnancy loss (RPL). The most widely accepted definition of RPL is two or three consecutive pregnancy losses, and it affects 1% of couples [2]. Risk factors for RPL include epidemiological, genetic, and endocrine factors; antiphospholipid syndrome; and anatomical disorders, including congenital malformation, intrauterine adhesions, fibroids, and cervical incompetence [3].

Cervical incompetence usually occurs in the second or early third trimester [4] and is defined, according to the inclusion criteria for the Cervical Incompetence Prevention Randomized Cerclage Trial (CIPRACT), as “*the initial, painless, progressive dilatation of the uterine cervix, where preterm birth seems inevitable without interference*” [5]. Cervical incompetence can be diagnosed in the absence of other causes of preterm birth, such as uterine anomaly, fibroids, or infection.

Cervical cerclage is an effective surgical procedure for the prevention of miscarriage due to cervical factors, and cervical incompetence, which is estimated to occur in about 1% of all pregnancies, is thus a common indication for cervical cerclage [6]. As it has been established that cervical cerclage can correct cervical incompetence [7], we explored the possibility that a laparoscopic cerclage procedure could interrupt RPL.

Transvaginal cerclage (TVC) was the first cervical cerclage and has been used to treat cervical incompetence since the 1950’s [8]. TVC is easily approached by the operator but is not suitable for patients with a history of failed transvaginal cerclage or an inability to undergo a transvaginal procedure because of an extremely short or absent cervix. Cervical cerclage with a transabdominal approach was first used as an alternative procedure in 1965 [9], but transabdominal cerclage can be a burden on the patient—postoperative adhesion can result from the laparotomy approach, and another laparotomy is required in the event of subsequent cesarean sections. Since laparoscopic transabdominal cerclage during pregnancy was first reported in 1996, cerclage using laparoscopy has replaced the transabdominal and vaginal approaches [10].

Our center has previously reported on the feasibility of modified laparoscopic transabdominal cervicoisthmic cerclage (LTCC) using tape inserted outside the uterine vessels and above the ureter, which is an easier and simpler method than conventional LTCC [11]. In this study, we evaluated whether modified LTCC is a feasible and safe surgical procedure based on 16 years of surgical experience at a single institution.

## 2. Materials and Methods

### 2.1. Data Source

A retrospective observational cohort study was conducted of patients who underwent modified LTCC over the 16-year period from 2003 to 2018 at Keimyung University Dongsan Hospital, Daegu, Korea. All procedures were performed by a single surgeon. The approval of the Institutional Review Board (IRB) was obtained to collect hospital records (Protocol number 07–26).

Indications for modified LTCC were (1) a history of second trimester losses or (2) severe defect of the cervix with or without a shortened cervix (less than 25mm) [12]. Detailed characteristics of each indication subgroup are shown in Table 1. Patients were operated upon regardless of underlying conditions that could complicate the surgical procedure.

There were no specific contraindications for modified LTCC, but it was necessary to preoperatively perform the following steps to ensure a safe procedure: (1) obtain endocervical swabs for chlamydia and mycoplasma to check for cervical infections, (2) monitor fetal viability and check cervical length by transvaginal ultrasonography, and (3) check for abdominal discomfort, ruptured membranes, or vaginal bleeding. Patients with positive endocervical swabs for chlamydia or mycoplasma received preoperative antibiotic treatment.

Prior to surgery, all patients who were to undergo modified LTCC were informed, via a written consent form, of the techniques used in the procedure, the benefits of a laparoscopic approach compared to laparotomy, the risk of complications related to the procedure, and the possibility of laparotomic conversion. The ages of the patients were recorded at the time of surgery, which was usually scheduled before the second trimester, considering the difficulty of the laparoscopic approach and the ease of uterine manipulation during surgery at this stage of pregnancy. Combined spinal and general anesthesia was performed to reduce the adverse effects of anesthesia on both the patient and fetus. The surgical procedure for modified LTCC with tape inserted outside the uterine vessels is described in our previous study, and all surgeries in this study followed that procedure [11]. The data collected on operative outcomes included operation time, preoperative and postoperative laboratory findings, operative complications, length of hospital stay, and indications for surgery.

The patients visited an outpatient clinic to confirm fetal viability and the location of tape placement by transvaginal ultrasonography two weeks after discharge. They were then followed by an obstetric team for prenatal care. The obstetric outcome data included gestational week at delivery, fetal survival rate, neonatal weight, and need for the neonatal intensive care unit (NICU). Deliveries were classified into three categories according to delivery time: before 24 weeks, between 24 and 37 weeks, and after 37 weeks. We recommended that cerclage bands not be removed during cesarean section, and the patients would thus return to the outpatient clinic when they succeeded in their next pregnancy with a cerclage band in situ.

### 2.2. Statistical Analysis

Descriptive analysis was conducted using SPSS (version 25.0; IBM SPSS, Inc., Chicago, IL, USA) on the data for the indications for surgery, patient characteristics, and surgical and obstetric outcomes.

## 3. Results

**Figure 1:**
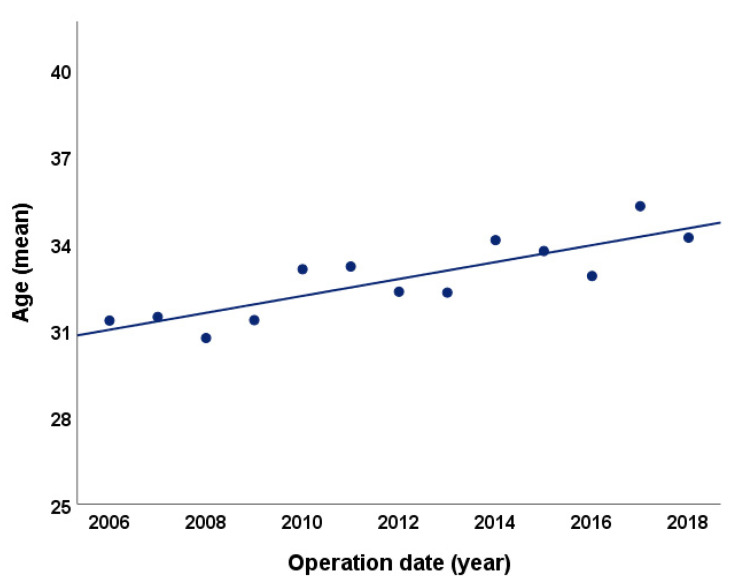
Mean age of patients by year.

A total of 299 patients underwent modified LTCC over the 16-year period from January 2003 to December 2018. The basic characteristics and surgical outcomes of the patients are described in Table 2. The mean patient age at the time of surgery was 32.3 years (range 22–43 years), and this increased during the study period Figure 1. The surgery was performed at a mean gestational age of 12.5 weeks (range 10.5–17.5 weeks). There were 80 cases of pregnancies through assisted reproductive technology (ART): four cases of ovulation induction (OI), 12 cases of intrauterine insemination (IUI), 63 cases of in vitro fertilization–embryo transfer (IVF-ET), and one case of intracytoplasmic sperm injection (ICSI). Among the 299 patients, 190 instances of abortion history were reported (one abortion: 91 (47.9%), two: 59 (31.1%), three or more: 40 (21.1%)) before the present pregnancy and prior to the surgery. For underlying conditions, 14 patients exhibited adenomyosis and 113 patients exhibited pelvic adhesion. Most patients with a previous operation history presented with adhesion of the omentum, and eight patients had severe adhesion due to endometriosis.

The mean operation time was 47.4 min (range 15–100 min), and there were no conversions to laparotomy. There was little hemoglobin change (mean drop 1.1 g/dL) due to minimal blood loss during surgery, and no intraoperative or postoperative complications were associated with surgery. The patients were discharged an average of 6.5 days (range 3–85 days) postoperatively; one patient remained in the hospital for 85 days due to her wish for a longer hospital stay because of her fear of miscarriage. All other patients were discharged within two weeks post-operation.

Among the 299 patients who underwent modified LTCC, 94 were followed up for four weeks after the operation until they moved to another hospital for prenatal care in their hometowns. The remaining 205 patients were followed up at our center, and their obstetric outcomes are described in Table 3. Regarding fetal viability, a failed case was defined as pregnancy loss before 24 weeks. A total of 176 patients delivered successfully via cesarean section, and the fetal survival rate was 85.9% (176/205). Of these, 116 patients delivered at term and 60 delivered preterm before 37 gestational weeks. Of the 60 preterm delivery patients, most 80%, (48/60) delivered between 32 and 37 weeks.

Preterm labor (58.3%, 33/60) was the most common cause of preterm delivery, followed by premature rupture of membrane (PROM) in 15 patients (25%) and preeclampsia in five patients (8.3%) Table 4. There were 23 newborns (13.1%, 23/176) admitted to the NICU after delivery, but no long-term sequelae were reported and 29 patients (14.1%, 29/205) delivered before 24 weeks without fetal viability. The most common causes of fetal loss were spontaneous abortion (16 cases), PROM (five cases), and infection, such as chorioamnionitis (four cases). Mean gestational age and weight at delivery were 37 weeks and 2678 g, respectively. The cerclage band was left in place during cesarean section, and 29 patients had a successful second pregnancy with the cerclage band in situ.

## 4. Discussions

RPL is a common, heterogeneous condition, with an estimated prevalence of 0.5–3.0% in women of reproductive age [13]. It has also been well established that a history of pregnancy loss is associated with an increased risk of preterm birth in subsequent pregnancies [14,15].

The cervical factor is important in preterm birth because delivery requires the cervix to soften and dilate [16]. Cervical incompetence is traditionally managed by cervical cerclage—a surgical procedure that provides mechanical reinforcement of the cervix [17]. This is a common procedure, although some studies have reported concerns about its efficacy and complications [18,19].

The laparoscopic approach is nowadays preferred by well-trained surgeons due to the advantages of shorter recovery times and hospital stays and less postoperative pain and adhesion formation than the open method. A study by Ades et al. of laparoscopic transabdominal cerclage during pregnancy reported that all 19 patients enrolled in this study had no operative complications and were discharged within one day [20].

We also previously reported a preliminary study that showed the feasibility of LTCC during pregnancy to address RPL in 80 patients, with a mean operation time of 52 min [11]. In that study, the patient’s condition—including the possibility of adhesion from a previous operation—was considered before selection for modified LTCC. Based on the successful outcomes, we extended our research to include patients regardless of underlying conditions that might complicate surgical procedures. The surgical and obstetric outcomes of our previous and current studies are comparable, despite the inclusion of patients with underlying conditions, and the comparability of the outcomes in the current study is, we believe, attributable to the improved proficiency of the surgeon, resulting in mean operation time being shortened from 52 min in the previous study to 47.4 min in the current study.

Previous studies have shown no significant differences in obstetric outcomes, regardless of the surgery method—laparotomy, laparoscopy, or robotic surgery [8,21,22]—although a recent study by Moawad et al. claimed improved obstetric outcomes associated with the laparoscopic approach [23]. The disadvantages of laparotomy are well reported, including longer operation time and hospital stays and greater postoperative adhesion than laparoscopy [8]. It also provides poor visibility from the surgeon’s perspective and requires additional laparotomies in event of subsequent cesarean deliveries. Furthermore, robotic surgery is not cost-effective in Korea, costing 20 times as much as laparoscopy; the laparoscopic approach is therefore favored over both laparotomy and robotic surgery [8,24].

The mean operation time in this study is shorter than that of other reports on laparoscopic and robotic transabdominal cerclage [21,24], which reduces the total dose of analgesic agents. Importantly, the negative effect of analgesic agents on fetuses depended on the dose administered [25], and combined spinal and general anesthesia was used in this study to further reduce the total dose.

Conization—a risk factor for RPL—is a surgical procedure essential for treating preinvasive cervical lesions and early cervical cancer (stage IA1). It preserves fertility but causes cervical damage and defects. The incidence of preinvasive cervical lesion and cervical cancer is increasing, especially in women of reproductive age [26]. As the rate of conization increases in young women, the risk of preterm labor–related post-conization states also increases. Because conization eliminates part of the cervix, second trimester pregnancy losses due to cervical incompetence from lack of mechanical support are worrisome [27]. Concern about preterm labor associated with conization is an obstacle to proper management of preinvasive cervical lesions and early cervical cancer in patients of reproductive age. In this study, patients with a history of cervical surgery—including conization—accounted for 34.8% (104/299) of the cases. As a surgical procedure, LTCC offers a favorable fetal survival rate (85.6%), including in cases with a history of cervical surgery. This suggests that miscarriage and preterm labor after conization could be prevented. 

This study is limited by the absence of a compatible control group and by losses to follow-up and thus obstetric outcomes. Another limitation is that the procedure was performed by only one experienced surgeon. Nevertheless. to our knowledge, this is the largest cohort study of patients who have undergone LTCC during pregnancy.

Most current laparoscopic TAC series’ report on a fetal survival of >90%, a fetal loss rate or miscarriage rate of <7% and a spontaneous preterm birth rate of at most 5–10% [8,23,24]. Yet, in this series the fetal survival rate is only 86% and is further confounded by a >10% miscarriage rate and >10% preterm birth rate. The relatively low fetal survival rate in this study is thought to be influenced by the size and composition of patient population. Many patients living in other regions or overseas visited only for surgery. They referred from obstetric team for LTCC because other surgical approaches such as transvaginal cerclage or conventional laparotomic transabdominal cerclage were considered unsuitable for them. All patients from nationwide were operated regardless of underlying conditions that could complicate the surgical procedure including adenomyosis, previous operation history and pelvic adhesion. Despite of relatively lower fetal survival rate compared to other laparoscopic TAC series’ report, the strengths of this study are unrestricted patient selection, the relatively large sample size and the consistency of the data, which offers promising obstetric outcomes.

The results suggest that modified LTCC during pregnancy is a safe and feasible surgical option for patients with a history of RPL, and especially RPL due to cervical incompetence. Modified LTCC may be considered surgically safe and effective, and its operation time, operative technique, and low risk of complications suggest positive outcomes. It also shows more favorable fetal survival rates and gestational ages at delivery than other techniques. This study therefore presents a standardized, well-established, and favorable surgical procedure that can be mastered with ease.

## Figures and Tables

**Table 1:** Surgical indication of modified LTCC.

Parameter	Number of Patients (*n* = 299)
** History of second trimester loss(es)**	190 (63.5%)
Failure of TVC	132 (69.5%)
Cervical defect with history of cervical surgery	4 (2.1%)
Cervical defect with history of failure of TVC and cervical surgery	17 (8.9%)
Cervical defect without history of failure of TVC and cervical surgery	37 (19.5%)
** Severe defect of cervix with or without shortened cervix**	109 (36. 5%)
Failure of TVC	24 (22.0%)
Cervical defect with history of cervical surgery	62 (56.9%)
Cervical defect with history of failure of TVC and cervical surgery	21 (19.3%)
Cervical defect without history of failure of TVC and cervical surgery	2 (1.8%)

Data are presented as number (%). Abbreviations: LTCC, laparoscopic transabdominal cervicoisthmic cerclage; TVC, transvaginal cerclage.

**Table 2:** Basic characteristics and surgical outcomes of modified LTCC.

Parameter	Modified LTCC (*n* = 299)	Subgroup	
		History of Second Trimester Loss (*n* = 190)	Severe Defect of Cervix with or without Shortened Cervix (*n* = 109)
** Age (years)**	32.3 (22–43)	32.5 (22–43)	32.2 (22-43)
<30	62 (20.7%)	40 (21.1–115%)	22 (20.2%)
30–35	185 (61.9%)	(60.5%)	70 (64.2%)
>35	52 (17.4%)	35 (18.4%)	17 (15.6%)
** ART**	80 (26.8%)	46 (24.2%)	34 (31.2%)
OI	4 (5.0%)	2 (4.3%)	2 (5.9%)
IUI	12 (15.0%)	6 (13.0%)	6 (17.6%)
IVF	63 (78.8%)	37 (80.4%)	26 (76.5%)
ICSI	1 (1.3%)	1 (2.2%)	0 (0%)
** Preterm delivery history**	185 (61.9%)	177 (93.2%)	8 (7.3%)
1	110 (59.5%)	103 (58.2%)	7 (87.5%)
2	58 (31.4%)	57 (32.2%)	1 (12.5%)
≥3	17 (9.2%)	17 (9.6%)	0 (0%)
** Abortion history**	190 (63.5%)	118 (62.1%)	72 (66.1%)
1	91 (47.9%)	53 (44.9%)	38 (52.8%)
2	59 (31.1%)	39 (33.1%)	20 (27.8%)
≥3	40 (21.1%)	26 (22.0%)	14 (19.4%)
** Gestational weeks at surgery**	12.5 (10.5–17.5)	12.3 (10.5–16.3)	13.1 (11.0–17.5)
** Twin pregnancy surgery**	36 (12.0%)	24 (12.6%)	12 (11.0%)
** Hemoglobin drop (g/dL)**	1.1 (0–3.4)	1.1 (0–−3.4)	1.2 (0–3.1)
** EBL (mL)**	70.1 (0–200)	71.1 (0–200)	68.6 (0–100)
** Operation time (minutes)**	47.4 (15–100)	47.4 (15–100)	47.2 (20–95)
** Operative complications**	0 (0%)	0 (0%)	0 (0%)
** Hospital stay (days)**	6.5 (3–85)	6.9 (3–85)	3.6 (3–30)
** Intraoperative pelvic adhesion**	113 (37.8%)	66 (34.7%)	47 (43.1%)
** History of cesarean section**	53 (17.7%)	37 (19.5%)	16 (14.7%)
** History of other abdominal surgery**	63 (21.1%)	37 (19.5%)	26 (23.9%)

Data are presented as mean (range) or number (%) d. Abbreviations: LTCC, Laparoscopic transabdominal cervicoisthmic cerclage; OI, ovulation induction; IUI, intrauterine insemination; IVF-ET, in vitro fertilization-embryo transfer; ICSI, intracytoplasmic sperm injection; EBL, estimated blood loss.

**Table 3:** Obstetrics outcomes of modified LTCC.

Parameter	Number of Patients (*n* = 205)	Subgroup	
		History of Second Trimester Loss (*n* = 134)	Severe defect of Cervix with or without Shortened Cervix (*n* = 71)
** Delivery at 13–23 + 6 weeks**	29 (14.1%)	17 (12.7%)	12 (16.9%)
Spontaneous abortion	16 (55.2%)	9 (52.9%)	7 (58.3%)
Premature rupture of membranes	5 (17.2%)	4 (23.5%)	1 (8.3%)
Infection	4 (13.8%)	1 (5.9%)	3 (25.0%)
Termination due to fetal anomaly	3 (10.3%)	2 (11.8%)	1 (8.3%)
Others	1 (3.4%)	1 (5.9%)	0 (0%)
** Preterm delivery (24–36 + 6weeks)**	60 (29.3%)	39 (29.1%)	21 (29.6%)
24–26 + 6 weeks	7 (11.7%)	6 (15.4%)	1 (4.8%)
27–31 + 6 weeks	5 (8.3%)	5 (12.8%)	0 (0%)
32–36 + 6 weeks	48 (80.0%)	28 (71.8%)	20 (95.2%)
** Term delivery (≥37 weeks)**	116 (56.6%)	78 (58.2%)	38 (53.5%)
** Fetal survival rate**	176 (85.9%)	117 (87.3%)	59 (83.1%)
Gestational weeks at delivery	37 (26–40)	36 (24–39)	37 (25–40)
Baby weight (g) (living newborn)	2678 (690–4100)	2680 (690–4100)	2673 (840–3540)
Low birth weight (< 2500 g)	50 (28.4%, (50/176))	33 (28.2%, (33/117))	17 (28.8%, (17/59))
Requiring NICU care	23 (13.1%, (23/176))	20 (17.1%, (20/117))	3 (5.1%, (3/59))
** Successful next pregnancy with cerclage band in situ**	29 (14.1%)	20 (14.9%)	9 (12.7%)

Data are presented as mean (range) or number (%). Abbreviations: LTCC, Laparoscopic transabdominal cervicoisthmic cerclage; NICU, Neonatal intensive care unit.

**Table 4:** Cause of preterm delivery after modified LTCC.

Cause of Preterm Delivery (24–36 + 6 Weeks)	Number of Patients (*n* = 60)	Subgroup	
		History of Second Trimester Loss (*n* = 39)	Severe Defect of Cervix with or without Shortened Cervix (*n* = 21)
** Preterm labor**	33 (55.0%)	21 (53.8%)	12 (57.1%)
24–26 + 6 weeks	2 (6.1%)	2 (9.5%)	0 (0%)
27–31 + 6 weeks	2 (6.1%)	2 (9.5%)	0 (0%)
32–36 + 6 weeks	29 (87.9%)	17 (81.0%	12 (100%)
** Preterm premature rupture of membrane**	15 (25.0%)	7 (17.9%)	8 (38.1%)
24–26 + 6 weeks	2 (13.3%)	1 (14.3%)	1 (12.5%)
27–31 + 6 weeks	2 (13.3%)	2 (28.6%)	0 (0%)
32–36 + 6 weeks	11 (73.3%)	4 (57.1%)	7 (87.5%)
** Preeclampsia**	5 (8.3%)	5 (12.8%)	0 (0%)
** Others**	7 (11.7%)	6 (15.4%)	1 (4.8%)

Data are presented as number (%). Abbreviations: LTCC, laparoscopic transabdominal cervicoisthmic cerclage.

## Data Availability

The data presented in this study are available on request from the corresponding author.
